# High quality RNA isolation from *Aedes aegypti *midguts using laser microdissection microscopy

**DOI:** 10.1186/1756-3305-4-83

**Published:** 2011-05-19

**Authors:** Young S Hong, Seokyoung Kang, Manjong Han, Geoffrey N Gobert, Malcolm K Jones

**Affiliations:** 1 Department of Tropical Medicine, School of Public Health and Tropical Medicine, Tulane University, New Orleans, Louisiana 70112, USA; 2 Department of Cell and Molecular Biology, School of Science and Engineering, Tulane University, New Orleans, Louisiana 70118, USA; 3 Queensland Institute of Medical Research, 300 Herston Road, Herston, Qld 4006, Australia; 4 School of Veterinary Sciences, The University of Queensland, Qld, 4072, Australia

## Abstract

**Background:**

Laser microdissection microscopy (LMM) has potential as a research tool because it allows precise excision of target tissues or cells from a complex biological specimen, and facilitates tissue-specific sample preparation. However, this method has not been used in mosquito vectors to date. To this end, we have developed an LMM method to isolate midgut RNA using *Aedes aegypti*.

**Results:**

Total RNA was isolated from *Ae. aegypti *midguts that were either fresh-frozen or fixed with histological fixatives. Generally, fresh-frozen tissue sections are a common source of quality LMM-derived RNA; however, our aim was to develop an LMM protocol that could inactivate pathogenic viruses by fixation, while simultaneously preserving RNA from arbovirus-infected mosquitoes. Three groups (10 - 15 mosquitoes per group) of female *Ae. aegypti *at 24 or 48-hours post-blood meal were intrathoracically injected with one of seven common fixatives (Bouin's, Carnoy's, Formoy's, Cal-Rite, 4% formalin, 10% neutral buffered formalin, or zinc formalin) to evaluate their effect on RNA quality. Total RNA was isolated from the fixed abdomens using a Trizol^® ^method. The results indicated that RNA from Carnoy's and Bouin's fixative samples was comparable to that of fresh frozen midguts (control) in duplicate experiments. When Carnoy's and Bouin's were used to fix the midguts for the LMM procedure, however, Carnoy's-fixed RNA clearly showed much less degradation than Bouin's-fixed RNA. In addition, a sample of 5 randomly chosen transcripts were amplified more efficiently using the Carnoy's treated LMM RNA than Bouin's-fixed RNA in quantitative real-time PCR (qRT-PCR) assays, suggesting there were more intact target mRNAs in the Carnoy's fixed RNA. The yields of total RNA ranged from 0.3 to 19.0 ng per ~3.0 × 10^6 ^μm^2 ^in the LMM procedure.

**Conclusions:**

Carnoy's fixative was found to be highly compatible with LMM, producing high quality RNA from *Ae. aegypti *midguts while inactivating viral pathogens. Our findings suggest that LMM in conjunction with Carnoy's fixation can be applied to studies in *Ae. aegypti *infected with arboviruses without compromising biosafety and RNA quality. This LMM method should be applicable to other mosquito vector studies.

## Background

The ability to isolate a specific type or a subpopulation of cells from tissue composed of heterogeneous cell types is highly desirable for many experimental investigations. A homogeneous sample will represent a more congruent cellular response to either biotic or abiotic stimuli compared to an admixture of different cell types. Use of homogeneous biological samples, therefore, is likely to enhance detection power of cellular changes in quantitative assays such as quantitative real-time PCR (qRT-PCR), microarrays, and proteome analyses [1-3]. In this regard, LMM has proven to be a powerful tool because it allows direct isolation of tissues and cells of interest based on morphology or tagging using laser-aided excision under a microscope [4,5]. While LMM has been widely utilized for molecular analyses of pathological samples from humans and other vertebrates and invertebrates [6,7], it has rarely been used in insects, with the exception of *Drosophila melanogaster *[8-11]. As mosquitoes play a key role in infectious disease transmission, development of an LMM method for mosquito vectors will facilitate gene expression profiling and lead to a better understanding of gene function relevant to disease transmission.

To perform LMM, fresh tissues may be embedded in a cryo-medium (e.g., O.C.T., Tissue-Tek, CA) by snap-freezing without fixation [5,8]. Rapid freezing of fresh tissues in a cryo-medium immediately after dissection helps preserve the quality of biological extracts such as RNA, DNAs, and proteins [5]. However, tissue samples are often fixed with a histological fixative and subsequently embedded in paraffin [5,12]. This fixation and embedding process can protect structural integrity of tissues for an extended period of time. Because visual identification of target regions is necessary for excision in tissues by microscopy, maintenance of cellular morphology by proper fixation is critical. However, some fixatives may have harmful effects on cellular extracts. In particular, cross-linking fixatives such as paraformaldehyde (or formalin) and glutaraldehyde have been reported to cause considerable degradation of RNA in tissues [13,14]. Despite this potentially detrimental undertaking, fixation may be absolutely necessary when mosquitoes are infected with infectious pathogens that require inactivation [15]. Therefore, caution needs to be taken in optimizing a method of fixation to obtain the best preservation of biological extracts for post-LMM analyses when infectious materials are processed. With the promising utility of LMM for mosquito vector research, we have established a practical LMM method utilizing Carnoy's, a commonly available fixative, for use in *Aedes aegypti*. In the present study, we have demonstrated the feasibility of the LMM method using *Ae. aegypti *as a model, and we believe that this method can be readily applied to other mosquito vectors.

## Methods

### Mosquitoes

The Rockefeller strain of *Aedes aegypti *was reared at 25°C and 80% relative humidity under a 12 h light: 12 h dark photoperiod in the laboratory following a general mosquito rearing guide [16]. Adult mosquitoes were supplied with a cotton ball soaked in a 10% sucrose solution *ad libitum *and fed on sheep blood meal (Hemostat Laboratories, Dixon, CA) for one hour using a glass membrane feeder (Lillie Glasswork, Atlanta, GA) once a week for egg production. The membrane feeder was kept at 37°C by circulating preheated water through a water bath during blood feeding. Larval mosquitoes were provided with 10% suspension of bovine liver powder (MP Biochemicals, Cat# ICN90039601) in distilled water.

### Fixation of Mosquitoes

Mosquitoes were blood-fed as described above to satiation for 1 hour and kept at 25°C and 80% relative humidity under a 12 h light: 12 h dark photoperiod in an environmental chamber (Model I66-VL, Percival Scientific, Perry, IA). At 24 or 48 hours post-blood meal, engorged female mosquitoes were immobilized and harvested by placing them in 2-3 minute cold-shock at -20°C. For fixation, each fixative was intrathoracically injected into the mosquito using a 30.5 gauge needle on a 1 ml syringe. The fixative-infused mosquitoes were kept on ice in a 1.5 ml microcentrifuge tube for 2 hours. After fixation, abdomens were dissected using a pair of forceps and immediately processed whole for RNA extraction (see below). Three groups (10 - 15 abdomens per group) were prepared for each of the following fixatives:

▪ Bouin's (Ricca Chemical, Cat# 1120)

▪ Carnoy's (10 ml glacial acetic acid, 30 ml chloroform, 60 ml 95% ethanol)

▪ Formoy's (10 ml acetic acid, 30 ml 40% formaldehyde, 60 ml 95% ethanol)

▪ Cal-Rite (Thermo Scientific, Cat# 5501)

▪ 4% formalin in PBS (pH 7.4, USB Chemicals Cat# 19943)

▪ 10% neutral buffered formalin (Thermo Scientific, Cat# 5701)

▪ Zinc Formalin (Thermo Scientific, Cat# 5701ZF).

### Cryosectioning and Laser Microdissection Microscopy (LMM)

After fixation with either Bouin's or Carnoy's fixative, 5-6 mosquito abdomens were dissected and embedded in the same orientation in O.C.T. Compound (Sakura Tissue-Tek, Torrence, CA) in aluminum foil cryomold (1.5 cm^3^). The embedded abdomens were snap-frozen by placing the cryomolds in an ethanol/dry ice bath. The embedded abdomens were prepared in triplicate and kept at -80°C until used. Laser microdissection was performed following the method described by Jones *et al*. [7] and Gobert *et al*. [17] with some modifications. Briefly, the embedded mosquitoes were cut across abdomens at 8 μm thickness with a knife pre-chilled to -25°C using a cryostat microtome (Leica Model CM3050 S). Before sectioning, the blade was cleansed with the RNAse Zap solution (Ambion, CA) to remove potential nuclease contamination. Mosquito abdomen sections were collected on adhesive membrane slides (P.A.L.M. Microlaser Technologies, Cat# 1440-1600). Each slide could accommodate 12-15 sections without overlapping. Following cryosectioning, samples were air-dried and kept in nuclease-free containers at room temperature until microdissection was performed.

Microdissection of mosquito midguts was performed using a P.A.L.M. laser microdissection system http://www.palm-microlaser.com equipped with a Zeiss Axiovert S100 microscope and its operating software (version 2.2). The cutting energy level of the laser beam was set to ~80% of the full output of the LMM unit. Microdissected midgut cells were captured onto PCR tube caps filled with adhesive gel (P.A.L.M. Microlaser Technologies, Cat# 1440-0260) and two independent collections for Bouin's and Carnoy's fixative were prepared for further analysis. Caps with dissected cells were placed in 200 μl PCR tubes stored at -80°C until RNA isolation.

### RNA isolation from LMM samples and frozen sections

Total RNA from laser-dissected tissues was isolated using the RNAqueous™ Micro kit according to manufacture's directions (Ambion, CA; Cat# 1931). This kit has been developed to optimize RNA isolation from LMM-cut tissues and cells. Briefly, 20 μl of lysis buffer was added into the LMM cap/PCR tube assembly and incubated for 30 min at 42°C with the caps positioned upside-down and touching the heating block. After incubation, the assembly was briefly centrifuged to collect the extraction fluid in the microcentrifuge tube. Then, an additional 80 μl of lysis buffer was added to a final volume of 100 μl. To each sample, 3 μl of the LMM Additive of the RNAqueous™ Micro kit was added, briefly mixed, and centrifuged. To recover total RNA, 1.25 × volumes (129 μl) of 95% ethanol was added to the samples and mixed by brief vortexing. Then, sample lysates were loaded onto spin columns, washed several times, and the total cellular RNA eluted twice each with 10 μl of elution buffer. To remove potential genomic DNA contaminants, the eluted RNA samples were treated with DNase I by adding 1/10 elution volume 10× DNase I buffer and 1 μl DNase I to each sample. After gentle but thorough mixing, the samples were incubated for 30 min at 37°C, after which DNase I reactions were stopped by adding 2 μl DNase Inactivation Reagent to each RNA sample followed by 2 min incubation at room temperature. The final RNA samples were stored at -20°C until use. As a control, total RNA was isolated from abdomens of five mosquitoes using TRIzol^® ^reagent http://www.invitrogen.com after snap-freezing without any fixation, as described above. The integrity of the RNA samples was assessed using Agilent RNA 6000 Pico Kit and an Agilent Bioanalyzer 2100 system according to the directions provided by the manufacturer http://www.chem.agilent.com.

### Quantitative Real-time polymerase chain reaction (qRT-PCR) assays

The integrity of RNA isolated from the LMM-cut midgut samples fixed with Carnoy's fixative and fresh-frozen midguts was compared to each other using qRT-PCR assays. For the qRT-PCR assays, there were four primer sets utilized to amplify five target genes (DQ440299, AAEL015246, AAEL012410, AAEL017251, and AAEL001466; Table 1). One primer set was able to amplify two independent mRNA (AAEL015246 and AAEL012410) in a gene family of translation elongation factors. First-strand cDNAs were synthesized in a final volume of 20 μl using SuperScript III™ reverse transcriptase (cat# 18080-051, Invitrogen, CA) following the manufacturer's instruction. In each reaction, 300 pg of total RNA in either 0.75 or 1.5 μl volume and 50 mM oligo(dT) primers were mixed with 1 μl of annealing buffer and the volume was adjusted with nuclease-free water to 8 μl. Then, the mixtures were incubated at 65°C for 5 min, immediately after which they were placed on ice for 1 min. To each sample, 2 μl SuperScript III reverse transcriptase and 10 μl 2× first-strand reaction mix (10 mM MgCl_2 _and 1 mM each dNTP) were added, making a final volume of 20 μl per reaction. Subsequently, the samples were incubated at 50°C for 50 min for reverse transcription and the reaction was terminated by heat inactivation at 85°C for 5 min and chilling on ice. The yields of first-strand cDNA synthesis were determined spectrophotometrically using a NanoDrop (ND1000, Thermo Scientific, PA). The cDNAs were kept at -80°C until use. Following fist-strand cDNA synthesis, real-time PCRs were performed using 0.7 μg first-strand cDNA in a total volume of 25 μl containing 12.5 μl of IQ™ SYBR Green Super Mix (cat# 170-8882, BioRad, CA) and 100 nM of each primer (Table 1) at the following thermocycling conditions: 95°C for 3 minutes, followed by 40 (DQ440299, AAEL015246, AAEL012410, and AAEL017251), or 55 (AAEL001466) cycles of denaturation at 95°C for 30 seconds and annealing/extension at 60°C for 1 minutes using an IQ5 real-time thermocycler (RioRad, CA). RNAs from two independent preparations were used in RT-PCR assays as biological replication.

**Table 1:** Primer sequences used to amplify the target transcripts from total RNAs isolated from LMM-cut *Aedes aegypti *midgut tissues.

Gene ID	Primer sequence (5' to 3')	Product size	Name
DQ440299	Forward: AACCAAGCAAACCCAAACC Reverse: AGGATACCGTTGGCATCG	195 bp	Heat shock cognate 70

AAEL015246 and AAEL012410	Forward:GCGGACGTAACTCATCCAC Reverse: CCCTGGTGACTGCAGAGATAG	498 bp	AGO1a and b

AAEL017251	Forward: TCAAGCAGACGAACCAAA Reverse: ATGGCACGAAGTTCTATGG	103 bp	AGO2

AAEL001466	Forward: TCTAACGCATACGCACCAC Reverse: TGTCGTCACCAGAGCAATG	130 bp	hypothetical protein

## Results

### Optimum Fixative for Preservation of Mosquito RNA

We tested seven different fixatives to examine their ability to preserve RNA quality following isolation from the mosquito abdomen harvested at 24 or 48-hr post-blood meal. These two time points were chosen because female mosquito midguts experience substantial physiological and mechanical changes such as stretching of midgut epithelial cells and induction of gene expression up to 48 hours after blood meal [18,19].

**Figure 1:**
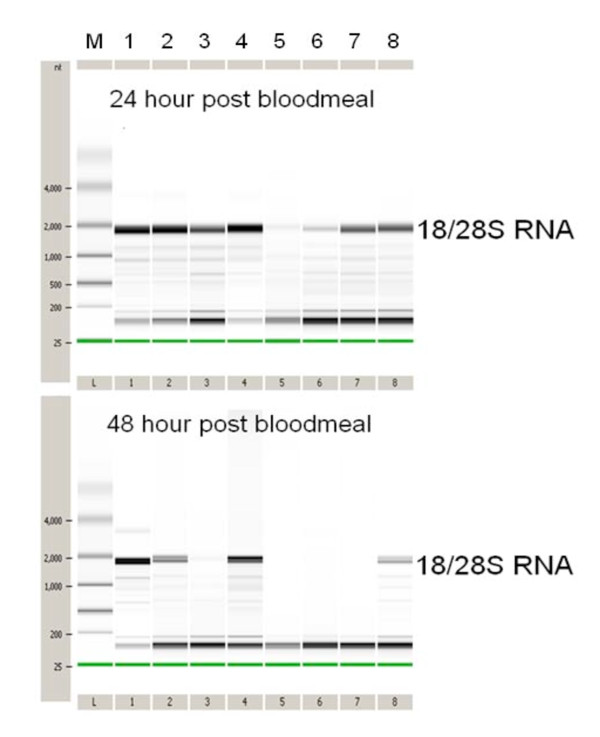
**Electrophoretic analysis of total RNA samples from the abdomen of adult female *Aedes aegypti *obtained using a Biolanalyzer 2100**. Adult mosquitoes were harvested at 24 (upper) or 48-hours (lower) post-blood meal and fixed with one of the following seven fixatives: 1) fresh frozen mosquitoes (control), 2) Bouin's, 3) Cal-Lite, 4) Carnoy's, 5) Formoy's, 6) NBF, 7) 4% paraformaldehyde, and 8) zinc formalin; M denotes size markers. In the 24-hour post-blood meal RNA, all but two, Formoy's and NBF, have intense discrete bands corresponding to 18/28S RNA that indicate quality RNA with minimal degradation. In the 48-hour post-blood meal samples, however, only Carnoy's, Bouin's, and zinc formalin-fixed samples display visible 18/28S RNA bands, suggesting these three fixatives preserve the mosquito RNA better than the other four fixatives can.

**Figure 2:**
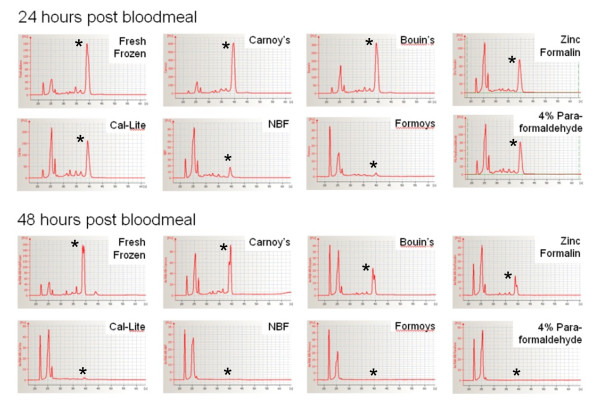
**Electropherograms of the *Aedes aegypti *RNA shown in Figure 1**. In support of Figure 1, RNA isolated from Carnoy's, Bouin's, or zinc formalin-fixed tissues maintain distinct peaks corresponding to 18/28S RNA marked by an asterisk (*). Carnoy's-fixed RNA has the largest peaks in both 24 and 48-hour post bloodmeal samples, and they are comparable to those of the control without fixation.

Therefore, midgut tissues at 24 and 28-hr post-blood meal present suitable stages to test the microdissection technique. In general, readily visible discrete ribosomal bands and an abundance of 18s/28s RNA bands (>2 kb) in Bioanalyzer analysis suggested high quality RNA (Figure 1). Most 24 hour post-blood meal samples had high quality RNA, except two RNA samples that had been fixed with NBF or Formoy's (Figure 1, lane 5 and 6). These two had notably faint 18/28S RNA bands and more intense small RNA products that were less than 100-bp, indicating significant degradation. For the 48 hour post-blood meal samples, however, only Carnoy's or Bouin's-fixed RNA (Figure 1, lane 2 and 4) were comparable to the control RNA extracted from fresh frozen mosquitoes. The remaining RNA samples had extensive degradation that lacked visible 18/28S bands (Figure 1). Electropherograms of the abdomen RNA further confirmed that Carnoy's and Bouin's fixatives yielded quality RNA similar to the control RNA after fixation. In contrast, the remaining 5 fixative samples had virtually no distinctive 18/28S peaks in 48 hour post-blood meal group. Only the zinc formalin-fixed RNA had visible 18/28S RNA bands; however, those peaks were much smaller than those from Carnoy's or Bouin's-fixed abdominal tissues (Figure 2).

### Laser Microdissection and RNA Extraction from Mosquito Midguts

**Figure 3:**
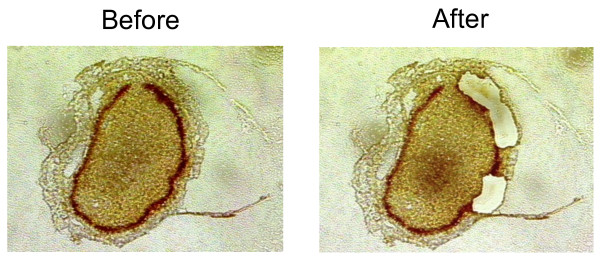
**Laser microdissection of *Aedes aegypti *midguts**. The abdomen from an adult female at 24-hour post-blood meal was fixed with Carnoy's fixative and cross-cut in 8 μm thickness (see text for the experimental details). The dark line is the midgut tissue and the hollow areas on the right panel are where the midgut tissues have been microdissected for RNA extraction by LMM.

**Figure 4:**
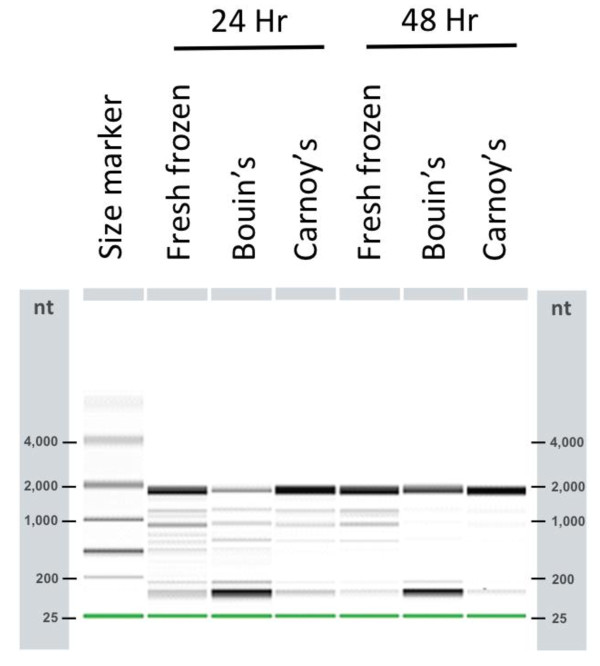
**Digital electrophoretic analysis of total RNA samples isolated from LMM-cut *Ae. aegypti *midgut tissues that were fixed with either Carnoy's or Bouin's fixative**. Total RNA from Carnoy's-fixed *Ae. aegypti *midguts shows strong high molecular weight bands around 2,000 bases and less visible low molecular weight bands of about 100 bases. Carnoy's-fixed RNA looks comparable to the control RNA that was prepared from freshly snap-frozen midguts without fixation. By contrast, Bouin's-fixed samples display more extensive degradation as seen in accumulation of low molecular weight bands of about 100 bp and weaker 2,000-base bands. Each RNA sample was isolated from microdissected midgut tissues from 10 - 15 midgut sections. Tissues from freshly frozen midguts were processed the same way as fixed samples and used as controls.

Based on the finding that Carnoy's and Bouin's fixatives did not appear to compromise *Ae. aegypti *RNA integrity, we further tested these two fixatives to evaluate their ability to preserve midgut RNA during the LMM procedure. Midgut tissues of *Ae. aegypti *fixed with either Bouin's or Carnoy's fixative were embedded in the O.C.T. medium, cryosectioned at 8 μm thickness and microdissected (Figure 3). Electrophoretic analyses of total RNA from the laser microdissected samples showed that the quality of Carnoy's-fixed RNA was far superior to Bouin's fixed RNA. Both 24 and 48-hour Bouin's-fixed samples showed extensive accumulation of small degraded RNA (Figure 4; see dark bands < 200 bases). By contrast, the high molecular weight bands were stronger in Carnoy's-fixed samples than in Bouin's-fixed samples, suggesting less RNA degradation in Carnoy's-fix RNA. In fact, the quality of Carnoy's-fixed RNA appeared to be comparable to that of fresh frozen controls, with no apparent degradation (Figure 4). The yields of total RNA from a batch of 10-15 microdissected sections of midgut tissues ranged from 0.3 to 19.0 ng (Table 2). Particularly, Carnoy's-fixed samples had 2.0 - 13.2 ng per ~3.0 × 10^6 ^μm^2^. In addition, the quality of the Carnoy's-fixed RNAs was further confirmed as the target mRNAs in the Carnoy's RNA samples were amplified as efficiently as in the control RNAs with comparable threshold cycle (Ct) values of qRT- PCR assays (Table 3).

**Table 2:** Yields of total RNAs prepared from microdissected *Aedes aegypti *midgut tissues.

		24 Hours Post Bloodmeal			48 Hours Post Bloodmeal		
		
Sample		Fresh frozen	Bouin's	Carnoy's	Fresh frozen	Bouin's	Carnoy's
RNA Conc. (pg/μl)	1*	883	806	244	952	515	659
	
	2	392	331	413	127	14	100

Total Yield (ng/20 μl)	1*	17.7	16.1	4.9	19.0	10.3	13.2
	
	2	7.8	6.6	8.3	2.5	0.3	2.0

Two independent preparations were performed. The samples denoted by an asterisk (*) are the ones, of which electrophoretic image are shown in Figure 4.

**Table 3:** Threshold cycle (Ct) values of quantitative real-time PCR (qRT-PCR) amplification.

		24 hour		48 hour	
Gene	Fixative	1^st^	2^nd^	1^st^	2^nd^
	Fresh	16.44	22.97	16.75	23.75
	
hsc70	Bouin's	19.50	32.45	22.49	27.44
	
	Carnoy's	16.98	16.34	16.59	17.20

	Fresh	27.32	34.87	26.22	34.94
	
ago1a & b	Bouin's	30.50	37.77	33.61	37.91
	
	Carnoy's	26.81	27.26	25.43	28.63

	Fresh	23.17	27.57	22.34	27.79
	
ago2	Bouin's	24.77	30.35	27.04	28.36
	
	Carnoy's	22.84	23.90	23.11	23.58

	Fresh	24.78	28.07	24.93	28.62
	
hypo. protein	Bouin's	25.66	33.36	31.70	29.27
	
	Carnoy's	24.43	24.94	26.00	27.03

Each target mRNA was amplified using primers listed in Table 1 and total RNAs prepared from microdissected *Aedes aegypti *midgut tissues in two independent preparations (1^st ^and 2^nd^).

### RNA Integrity and Fidelity

**Figure 5:**
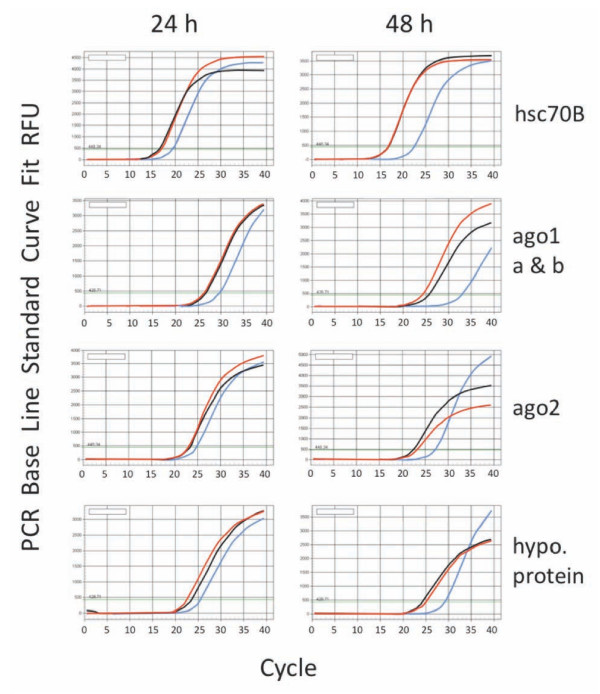
**Amplification of target mRNA by real-time PCR (RT-PCR) assays**. Total RNA isolated from laser microdissected tissues of Carnoy's-fixed (red lines), Bouin's (blue), or fresh snap-frozen (black lines) midguts of *Aedes aegypti *was used as templates in the RT-PCR assays in duplicate. The above are representative results of the duplicate assay.

Integrity of the RNA isolated from microdissected midgut tissues fixed with Bouin's or Carnoy's fixative was further examined by quantitative RT-PCR amplification of four randomly selected target mRNA. Initial reverse transcription reactions were performed using 300 pg total RNA as templates followed by PCR amplification of 4 sets of primers targeting 5 transcripts using 0.7 μg first-strand cDNA from the reverse transcription reactions. The amplification kinetics of target mRNAs from Carnoy's-fixed total RNA were comparable to those of the control RNA samples isolated from snap-frozen midgut sections (Figure 5). By contrast, the four target mRNAs were less abundant in total RNA samples isolated from Bouin's-fixed midgut tissues, as the linear amplification phase started later than the Carnoy's or control RNA (Figure 5). These results suggest that there are more amplifiable target mRNAs from the Carnoy's-fixed midguts than Bouin's-fixed midguts per unit total RNA. Therefore, high quality RNA can be prepared using an LMM method in *Ae. aegypti*, and isolated RNA is suitable for various molecular applications such as gene expression studies using microarray or RNA-Seq analyses.

## Discussion

Tissue or cell-specific preparation of biological extracts such as nucleic acids and proteins is critical for detecting cellular changes leading to mRNA or protein modulations. For example, viral infections in the mosquito midgut often are not systemic but form isolated infection foci [20]. If entire mosquitoes or abdomens are used for testing, uninfected cells will overwhelmingly outnumber virus-infected cells, thus masking or diluting transcriptional changes in virus-infected cells. Using *Ae. aegypti *midguts as a model, we have demonstrated that an LMM method can be effectively employed to prepare high quality total RNA in a tissue-specific manner. We were able to isolate high quality RNA from snap-frozen *Ae. aegypti *midguts that was also used as a control throughout the present study. However, as mosquitoes are vectors of many infectious pathogens, infected samples may require a histological fixative to inactivate pathogens for biosafety reasons.

We set out to test seven commonly used histological fixatives to evaluate their effects on RNA integrity of *Ae. aegypti *midguts. Among the tested fixatives, Carnoy's fixative RNA had the most prominent 18/28S bands and showed minimal accumulation of small RNA species of degradation, thus providing the best preservation of total RNA and virus inactivation for use in the LMM procedure. The acidic Carnoy's fixative quenches fluorescence from green fluorescent protein (GFP) that is widely used for protein tagging, but this quenching is reversible by 30 min washing in borate buffer (pH 8.5) and subsequent overnight incubation in phosphate-buffered saline (PBS, pH 7.2) at 4°C [21,22]. Consequently, it may be possible that Carnoy's fixative can be used to microdissect tissues or cells tagged with a GFP marker.

Molecular integrity of Carnoy's-fixed LMM RNA was evaluated using qRT-PCR of five test transcripts. Amplification kinetics of five target transcripts using the four primer sets (Table 1) suggested that there was no systematic reduction of target mRNA in Carnoy's-fixed samples compared to the control (Figure 5). This suggests that LMM-prepared RNA from Carnoy's-fixed *Ae. aegypti *midgut tissues was as intact as the fresh frozen control RNA. The yields of RNA prepared by LMM were estimated to range between 0.3 - 19.0 ng (median of 18.4 ng) per batch of 10 - 15 sections from control, Bouin's, or Carnoy's-fixed midguts. Based on the requirement of 5 - 200 ng of total RNA for an Agilent microarray hybridization [23], the RNA yields obtained by LMM in the present study should be sufficient for the Agilent oligomicroarray experiments in *Ae. aegypti *if several batches of LMM-prepared RNA are pooled together as reported in helminth LMM/microarray studies [17]. In addition, pooling RNA from several individual mosquitoes may have the advantage of normalizing genetic backgrounds that individual mosquitoes might have. In addition to the common histological fixatives tested in this study, fomalin-free commercial fixatives such as Molecular Fixative (Tissue-Tek, CA) and HOPE Fixative (Polysciences, PA) are available [24,25]. These fixatives are reported to maintain the integrity of both nucleic acids and proteins in pathological samples. However, these commercial fixatives are expensive compared to Carnoy's, which can be prepared freshly and inexpensively in any laboratory. More importantly, the quality of Carnoy's-fixed LMM RNA from *Ae. aegypti *midguts was exceptionally high, and target mRNA was also successfully amplified in qRT-PCR assays. For protein studies, we have yet to test the suitability of Carnoy's-fixed proteins in immunological (e.g., ELISA and Western blotting) or other proteomic assays. Nevertheless, preparation of sufficient quantities of proteins for proteomic studies would involve substantial LMM isolation because unlike nucleic acids, proteins cannot be amplified post-isolation.

In conclusion, we have demonstrated that tissue- or cell-specific isolation of *Ae. aegypti *RNA using LMM is feasible and that the RNA harvested using this method is of desirable quantity and quality for subsequent molecular assays. Development of this microdissection method should facilitate identification of candidate genes that play critical roles in infection mediation and pathogenesis of dengue virus in *Ae. aegypti*. This LMM method could be directly applicable to other major mosquito vectors such as *Anopheles gambiae *and *Culex *mosquitoes. Therefore, the utility of the LMM method is promising in vector biology research.

## Competing interests

The authors declare that they have no competing interests.

## Authors' contributions

YSH designed the experiment and performed the histology work, LMM, RNA isolation, Bioanalyzer assays, data analyses, and wrote the manuscript. SK and MH performed qRT-PCR and cryosectioning, respectively. GNG and MKJ assisted LMM and reviewed the manuscript.
